# Association between red cell distribution width to albumin ratio and all-cause mortality in patients with acute pancreatitis admitted to the intensive care unit: a retrospective study based on the MIMIC-IV database

**DOI:** 10.3389/fmed.2025.1503378

**Published:** 2025-02-12

**Authors:** Xuan Chen, Yuchen Luo, Side Liu

**Affiliations:** Guangdong Provincial Key Laboratory of Gastroenterology, Department of Gastroenterology, Nanfang Hospital, Southern Medical University, Guangzhou, Guangdong, China

**Keywords:** red blood cell volume distribution width to albumin ratio, acute pancreatitis, all-cause mortality, MIMIC-IV, serum biomarker

## Abstract

**Background:**

Red blood cell volume distribution width (RDW) and albumin (Alb) have been proved to be predictors of mortality in various diseases, such as acute pancreatitis (AP). However, until now the relationship between RDW to Alb ratio (RAR) and mortality in AP has not been fully elucidated. Therefore, this study aims to evaluate the relationship between RAR and all-cause mortality in AP.

**Method:**

Patients with AP in the Critical Care Medical Information Market (MIMIC-IV) database who met criteria were included in this retrospective study. Associated baseline data was obtained, cleaned and analyzed. Kaplan Meier (K-M) survival curve and Cox proportional hazards regression model were utilized to evaluate the relationship between RAR and all-cause mortality. Restricted Cubic Spline (RCS) was used for exploring how hazard ratio (HR) changes as RAR varied. Additionally, Receiver Operating Characteristic (ROC) analysis and subgroup analysis were conducted to assess the predictive value and to explore the significance of RAR in different populations.

**Results:**

499 patients were included in this study. Survival curve showed that patients with RAR > 5.14 had higher mortality rate at 7-day (d), 14-d, 21-d, 28-d, 90-d, 180-d and 1-year (y). The univariate and multivariate Cox models revealed an independent association between high-level RAR and all-cause mortality at 28-d, 90-d and 1-y. RCS showed that RAR became a risk factor when exceeding 5.14. RAR only had linear relationship with mortality at 1-y after adjusting for the potential confounders. Subgroup analysis suggested that increased RAR caused higher risk of death in male, non-white people or those patients without respiratory failure (RF). ROC analysis indicated that compared with other parameters such as SOFA score, RAR exhibited higher efficiency in predicting in-hospital and all-cause mortality at 14-d, 21-d, 28-d, 90-d. Combined RAR with BISAP, RAR-modified BISAP showed superiority in predicting short-term mortality (28-d).

**Conclusion:**

For patients with AP in ICU, RAR has a strong association with short- and long-term prognosis. Especially, RAR is a promising indicator for short-term all-cause mortality in patients with AP. For males, non-white patients and those without RF, elevated RAR may be a more dangerous signal of mortality.

## 1 Introduction

Acute pancreatitis (AP) is a common digestive system disease, with an annual incidence rate of about 13–45/100,000, of which gallstones, alcoholism and triglyceridemia are the main causes (1). Manifested as upper or total abdominal pain, bloating, and vomiting, most patients have mild symptoms, which can be controlled through supportive care and intravenous infusion therapy (1, 2). Accompanied by pancreatic necrosis and necrosis of pancreatic surrounding tissues, and even multiple organ failure, about 20% of patients develop moderate severe AP (MSAP) or severe AP (SAP) with the mortality rate up to 30% (3, 4).

Nowadays, there are several widely used scoring systems of assessing disease severity of AP: Sequential Organ Failure Assessment (SOFA), Acute Physiology and Chronic Health Evaluation II (APAChE-II), the Ranson criteria, and the Bedside Index for Severity in AP (BISAP) (5–8). However, these approaches are too complex to distinguish severe AP from mild AP early at the admission. For example, APAChE-II system requires a total of 14 parameters including age, chronic health status, and 12 other acute physiological markers (7). Also, SOFA system requires dynamic assessment of functional status of six systems consisting of respiration, coagulation, liver, circulation, nerves, and kidneys (6). Therefore, these evaluation systems require time to collect enough data in order to make accurate assessments, which may cause a delay in conducting optimal treatments. Thus, it is urgent and pivotal to develop a simpler and quicker indicator to assess AP severity timely.

Previously, some observational studies have demonstrated a relationship between prognosis and disease severity of AP and levels of blood markers such as creatinine (9), C-reactive protein (CRP) (10) and albumin (Alb) (11). When used for predicting AP severity and mortality, blood parameters like albumin are unsatisfying because of patients’ complicated pathophysiological state. Despite their poor predictive performance when used individually, combining multiple serum indicators can still enhances predictive power. For instance, according to Kaplan et al. (12), CRP/Alb >16.28 is an ideal predictor of mortality in AP with sensitivity and specificity reaching 92.1 and 58.0%, respectively. Red blood cell distribution width (RDW) is a parameter that reflects the heterogeneity of red blood cell volume size, often regarded as the coefficient of variation of red blood cell volume size (13). Recently, some retrospective studies have revealed that RDW and its derivative indicator are promising markers for the assessment of AP (14). According to He et al. (15), RDW, with area under curve (AUC) comparable to BISAP, is an independent risk factor for in-hospital mortality in patients with AP. As a derivative indicator, when used for determining severity of AP, RDW to Alb ratio (RAR) has a higher AUC (0.909) than RDW (AUC: 0.856), Alb (AUC: 0.864) or BISAP (AUC: 0.874) alone (16). Interestingly, combining RAR with BISAP, the AUC can reach 0.947 (16). Pan et al. (17) have developed a novel nomogram consisting of RAR and other eight parameters, which can predict 30-day mortality of patients with AP. In another observational study including 301 patients, RAR can help predict the occurrence of severe AP with an 80.0% sensitivity and an 80.7% specificity (14).

However, the relationship between RAR, as an individual indicator, and short- and long-term all-cause mortality in patients with AP remains obscure. Thus, we used the Medical Information Mart for Intensive Care IV version 3.0 [MIMIC-IV (v3.0)] database to collect, clean and analyze data of patients with AP admitted between 2008 and 2022. The study aimed to elucidate the association between short- and long-term all-cause mortality and RAR in patients with AP.

## 2 Methods

### 2.1 Data source

Created by the MIT Computational Physiology Laboratory, the MIMIC-IV (v3.0) database^1^ is a large, publicly available database, which provides all the data used in this study (18). MIMIC-IV database extensively collects specific data of Intensive Care Unit (ICU) and therefore has been an invaluable resource for clinical decision support, data modeling, critical care outcomes, and related field research. All patients admitted to the Beth Israel Deaconess Medical Center between 2008 and 2022 were included in this retrospective observational study. All the laboratory results, medications, operations, vital signs, length of hospital stay, and other specific information for patients were recorded. As all personal data in MIMIC-IV is de-identified and patient identification is replaced with random number, there is no need for ethical approval and informed consent. To obtain the qualification of using this database, the first author of this study, Xuan Chen, completed the Collaborative Institutional Training Initiative (CITI) course and passed both the “Conflicts of Interest” and “Data or Specimens Only Research” exams (Record ID: 63153641).

### 2.2 Criteria for population selection and results

**Figure 1:**
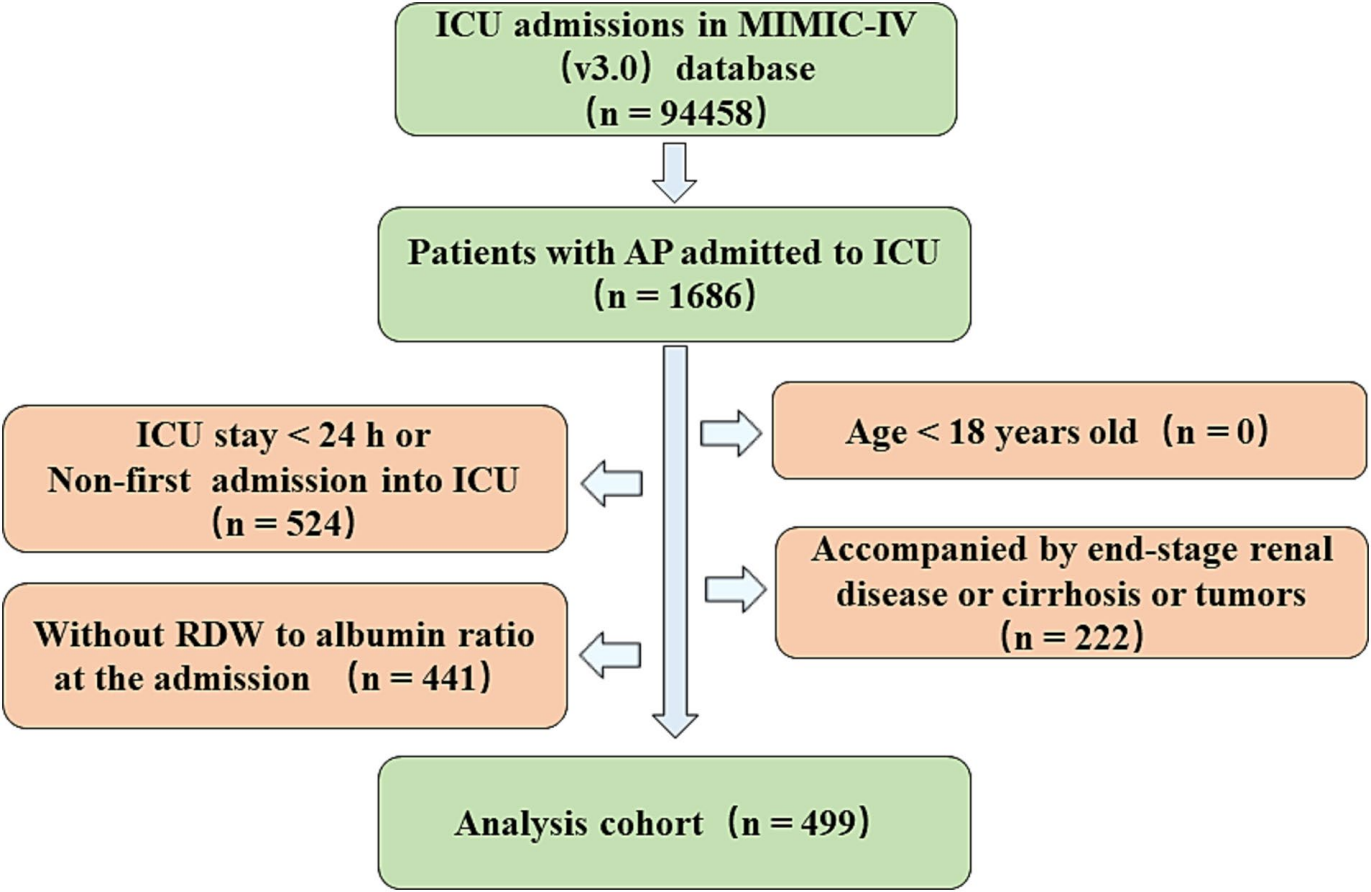
Flowchart for screening patients with AP from the MIMIC-IV (v3.0).

The MIMIC-IV database collects data on 94,458 ICU admissions between 2008 and 2022. Hospitalization data for patients with AP is screened using the International Classification of Diseases, 9th Revision (ICD-9) code 577.0, or International Classification of Diseases, 10th Revision (ICD-10) codes K85-K85.92. There was a total of 7,481 admissions with diagnosis of AP, of which 1,686 were ICU admission with AP as the first or other diagnosis. The patients with following characteristics were excluded in this study: those who were younger than 18 years old; those who stayed in ICU less than 24 h; those who were admitted into ICU repeatedly, only retain the data of first admission; those who had end-stage renal diseases, cirrhosis or tumors; and those whose RDW or Alb data was not recorded in the first 24 h of ICU stay. Finally, there were a total of 499 patients included in the study (Figure 1). None of the included patients received the potentially relevant medical treatments (red blood cells transfusion, human albumin infusion or parenteral nutrition) before ICU admission.

### 2.3 Data extraction

RAR was the main variable in this study. To minimize treatment-related interference, the first RDW and serum Alb within 24 h after admission to ICU were extracted. All the variables were extracted using the Structured Query Language (SQL). PostgreSQL was an openly available software used for installation and management of the database. Extraction procedure was consisting of 5 main components: demographic information, laboratory results, comorbidities, clinical treatments and clinical outcomes.

### 2.4 Endpoint events

All-cause mortalities at 7-day (d), 14-day (d), 28-day (d), 90-day (d), 180-day (d), 1-year (y) were set to be the primary outcome. In addition, we also analyzed the total length of hospitalization, the duration of ICU hospitalization, recurrence of ICU admission and in-hospital mortality.

### 2.5 Data cleaning

Variables with more than 15% missing values were excluded, such as amylase, lipase, lactate, high density lipoprotein, low density lipoprotein, total cholesterol, total triglyceride, basophils count, eosinophils count, lymphocytes count and monocytes count. Only one variable, prothrombin time (PT), which had missing values more than 5% but less than 15%, was processed using multiple interpolations (MI). The variables which had less than 5% missing values, including alkaline phosphatase (ALP), alanine aminotransferase (ALT), aspartate transaminase (AST), blood urea nitrogen (BUN), blood calcium, creatinine, Glasgow Coma Scale (GCS) score, blood glucose, SOFA score, blood sodium and total bilirubin (TB), were replaced with mean or median of that variable according to the result of normality test. Extreme values were handled by the Winsorize method, using 99 and 1% as cutoff points. The procedures mentioned above were conducted based on the software R (version 4.3.2).

### 2.6 Statistical analysis

Kolmogorov–Smirnov test was conducted to assess the normality of continuous variables. Continuous variables were expressed as mean ± standard deviation (SD) for normally distributed continuous variables, median (interquartile range) for non-normally distributed ones and numbers (%) for the categorical ones. When analyzing baseline characteristics, T test or One-Way ANOVA was used for comparing continuous variables, while Pearson’s χ^2^ test or Fisher’s test was used for comparing categorical variables. According to the median value of RAR, the 499 patients were divided into high-RAR group and low-RAR group. Survival curves were plotted via Kaplan–Meier (KM) method and *p*-values were calculated using Log-Rank test. Potential risk factors were screened using univariate Cox Regression analysis, and variables with p-value less than 0.1 were included in subsequent multivariate Cox Regression analysis. Restricted Cubic Spline (RCS) was used for analyzing non-linearity of RAR. To test whether RAR has any effect in various subgroups, subgroup analysis was completed and results were presented as forest plots. Finally, receiver Operating Characteristic (ROC) analysis was performed to assess the predictive power of RAR, RDW, Alb, SOFA, Systemic Inflammatory Response Syndrome (SIRS) score and Glasgow Coma Scale (GCS) and to calculate the AUC and the 95% confidence interval (CI) of each index. All the above analysis was done using software R (version 4.3.2) and the related packages. A two-tailed *p*-value less than 0.05 indicated statistical significance.

## 3 Results

### 3.1 Baseline demographic and clinical characteristics

Finally, a cohort of 499 patients with AP admitted into ICU were included in this study (Figure 1). The median age of 499 patients was 58, of whom 272 (55.51%) were male and 227 (44.49%) were female. Median of RAR was regarded as the cutoff value to divide patients into high-RAR group (>5.14) and low-RAR group (<=5.14). Compared with low-RAR group, high-RAR group had less male patients, higher SOFA score, higher white blood cell count (WBC), lower red blood cell count (RBC), higher platelet count, lower hemoglobin (Hb) concentration, lower hematocrit (Hct), higher BUN, lower blood calcium concentration and lower ALT. In terms of etiological background (alcoholic, biliary, drug-induced and unspecified), there was a similar proportion of composition between high-RAR group and low-RAR group, and the difference was not statistically significant. In terms of treatment, high-RAR group had higher usage rate of vasopressin, mechanical ventilation (MV) but lower usage rate of Endoscopic Retrograde Cholangiopancreatography (ERCP). In terms of comorbidity, compared to low-RAR group, high-RAR group tended to have higher incidence rate of acute kidney injury (AKI), Sepsis and respiratory failure (RF) but lower probability of being accompanied by hypertension. Meanwhile, the difference on the length of pre-ICU stay between the two groups was also not statistically significant. When it came to the clinical outcomes, high-RAR group had higher in-hospital mortality (19.28% vs. 6.80%, *p* < 0.001), higher incidence rate of multiple ICU admission (19.28% vs. 6.80%, *p* < 0.001), longer length of hospitalization (16.89 vs. 10.65, *p* = 0.004) and that of ICU stay (4.66 vs. 3.12, *p* = 0.010). In addition, the patients in high-RAR group had higher 7-d mortality (7.23% vs. 3.20%, *p* = 0.043), 14-d mortality (10.84% vs. 4.00%, *p* = 0.004), 21-d mortality (14.46% vs. 4.80%, *p* < 0.001), 28-d mortality (17.67% vs. 5.60%, *p* < 0.001), 90-d mortality (25.70% vs. 11.60%, *p* < 0.001), 180-d mortality (26.51% vs. 14.00%, *p* < 0.001) and 1-y mortality (28.51% vs. 17.20%, *p* = 0.003). Furthermore, compared with low-RAR group, high-RAR group had longer length of total hospital stay (16.89 vs. 10.65, *p* = 0.004) and longer ICU hospitalization (4.66 vs. 3.12, *p* = 0.010). The detailed results can be seen in the Table 1.

**Table 1:** Baseline characteristics in patients with AP.

Variables	Total (*N* = 648)	RAR level		*p* value
		RAR > 5.14 (*N* = 249)	RAR ≤ 5.14 (*N* = 250)	
Demographics				
Age (year)	58.0 (44.5, 71.0)	60.0 (46.0, 73.0)	55.5 (41.0, 70.0)	0.073
Gender (n, %)				0.022
Male	272 (55.51)	123 (49.40)	149 (59.60)	
Female	227 (45.49)	126 (50.60)	101 (40.40)	
Race (n, %)				0.813
White people	307 (61.52)	151 (60.64)	156 (62.40)	
Black people	44 (8.82)	21 (8.43)	23 (9.20)	
Other	148 (29.66)	77 (30.92)	71 (28.40)	
Etiology (%)				0.392
Alcoholic (%)	44 (8.82)	27 (10.84)	17 (6.80)	
Biliary (%)	39 (7.82)	18 (7.23)	21 (8.40)	
Drug-induced (%)	5 (1.00)	3 (1.20)	2 (0.80)	
Unspecified (%)	411 (82.36)	201 (80.72)	210 (84.00)	
SOFA score	6 (3, 10)	7 (4, 11)	5 (3, 9)	<0.001
GCS score	15 (15, 15)	15 (15, 15)	15 (15, 15)	0.937
Clinical treatments				
Octreotide (%)	28 (5.61)	19 (7.63)	9 (3.60)	0.050
Vasopressin (%)	79 (15.83)	53 (21.29)	26 (10.40)	<0.001
Betablockers (%)	201 (40.28)	98 (39.36)	103 (41.20)	0.675
MV (%)	279 (55.91)	163 (65.46)	116 (46.40)	<0.001
CRRT (%)	68 (13.63)	40 (16.06)	28 (11.20)	0.113
ERCP (%)	55 (11.02)	19 (7.63)	36 (14.40)	0.016
Comorbidities				
AKI (%)	342 (68.54)	192 (77.11)	150 (60.00)	<0.001
Sepsis (%)	351 (70.34)	188 (75.50)	163 (65.20)	0.012
RF (%)	231 (46.29)	130 (52.21)	101 (40.40)	0.008
HF (%)	82 (16.43)	35 (14.06)	47 (18.80)	0.153
AF (%)	101 (20.24)	53 (21.29)	48 (19.20)	0.562
Hypertension (%)	242 (48.50)	114 (45.78)	128 (51.20)	0.041
Diabetes (%)	162 (32.46)	78 (31.33)	84 (33.60)	0.587
Obesity (%)	79 (15.83)	40 (16.06)	39 (15.60)	0.887
Laboratory parameters				
WBC (10^9^/mL)	13.50 (9.50, 19.00)	14.40 (9.50, 19.00)	12.45 (9.60, 17.65)	0.036
RBC (10^12^/mL)	3.73 (3.22, 4.30)	3.47 (3.02, 4.12)	3.90 (3.44, 4.48)	<0.001
Plt (10^9^/mL)	199.00 (140.50, 278.00)	203.00 (148.00, 296.00)	193.50 (136.25, 261.00)	0.013
Hb (g/dL)	11.30 (9.70, 13.10)	10.40 (8.90, 12.20)	12.10 (10.70, 13.80)	<0.001
Hct (%)	34.40 (29.65, 39.50)	32.00 (28.00, 37.10)	36.00 (32.20, 41.80)	<0.001
PT (s)	14.40 (13.05, 16.80)	14.70 (13.30,17.10)	14.20 (12.90, 16.38)	0.641
Cr (mg/dL)	1.10 (0.70, 1.90)	1.10 (0.70, 2.30)	1.00 (0.70, 1.60)	0.053
BUN (mg/dL)	21.00 (12.00, 35.00)	23.00 (13.00, 40.00)	18.00 (11.00, 30.00)	0.001
Glucose (mg/dL)	140.50 (106.00, 189.00)	138.00 (106.00, 181.00)	143.00 (106.00, 202.75)	0.427
Calcium (mg/dL)	7.90 (7.30, 8.50)	7.70 (7.10, 8.30)	8.10 (7.40, 8.60)	<0.001
Potassium (mmol/L)	4.1 (3.6, 4.6)	4.0 (3.6, 4.5)	4.1 (3.6, 4.6)	0.536
Sodium (mmol/L)	138 (135, 141)	138 (134, 142)	138 (136, 141)	0.606
ALP (U/mL)	94.5 (65.0, 163.50)	98.0 (70.0, 172.0)	90.0 (62.25, 156.75)	0.058
ALT (U/mL)	49.50 (24.00, 145.50)	40.00 (23.00, 106.00)	66.00 (25.25, 186.00)	0.033
AST (U/mL)	70.00 (34.00, 148.00)	57.00 (32.00, 129.00)	86.50 (38.25, 181.00)	0.163
TB (mg/dL)	0.90 (0.60, 2.20)	0.80 (0.50, 1.70)	1.00 (0.60, 2.78)	0.068
Clinical outcomes				
LOS pre-ICU (hour)	1.01 (0.02, 2.91)	1.02 (0.02, 6.82)	1.01 (0.02, 2.32)	0.469
LOS ICU (day)	3.67 (1.95, 9.21)	4.66 (2.34, 12.64)	3.12 (1.85, 6.66)	0.010
LOS Hospital (day)	13.16 (7.27, 23.31)	16.89 (9.15, 27.60)	10.65 (5.89, 19.77)	0.004
In-hospital mortality (%)	65 (13.03)	48 (19.28)	17 (6.80)	<0.001
ICU re-admission (%)	65 (13.03)	41 (16.47)	24 (9.60)	0.023
7-day mortality (%)	26 (5.21)	18 (7.23)	8 (3.20)	0.043
14-day mortality (%)	37 (7.41)	27 (10.84)	10 (4.00)	0.004
21-day mortality (%)	48 (9.62)	36 (14.46)	12 (4.80)	<0.001
28-day mortality (%)	58 (11.62)	44 (17.67)	14 (5.60)	<0.001
90-day mortality (%)	93 (18.64)	64 (25.70)	29 (11.60)	<0.001
180-day mortality (%)	101 (20.24)	66 (26.51)	35 (14.00)	<0.001
365-day mortality (%)	114 (22.85)	71 (28.51)	43 (17.20)	0.003

SOFA, Sequential Organ Failure Assessment; GCS, Glasgow Coma Scale; MV, mechanical ventilation; CRRT, continuous renal replacement treatment; ERCP, Endoscopic Retrograde Cholangiopancreatography; AKI, acute kidney injury; RF, respiratory failure; HF, heart failure; AF, atrial fibrillation; WBC, white blood cell count; RBC, red blood cell count; Plt, platelet; Hb, hemoglobin; Hct, hematocrit; PT, prothrombin time; Cr, creatinine; BUN, blood urea nitrogen; ALP, alkaline phosphatase; ALT, alanine aminotransferase; AST, aspartate aminotransferase; TB, total bilirubin; LOS ICU, length of ICU stay; LOS Hospital, length of hospital stay.

### 3.2 Kaplan–Meier survival curve and cox regression analysis

**Figure 2:**
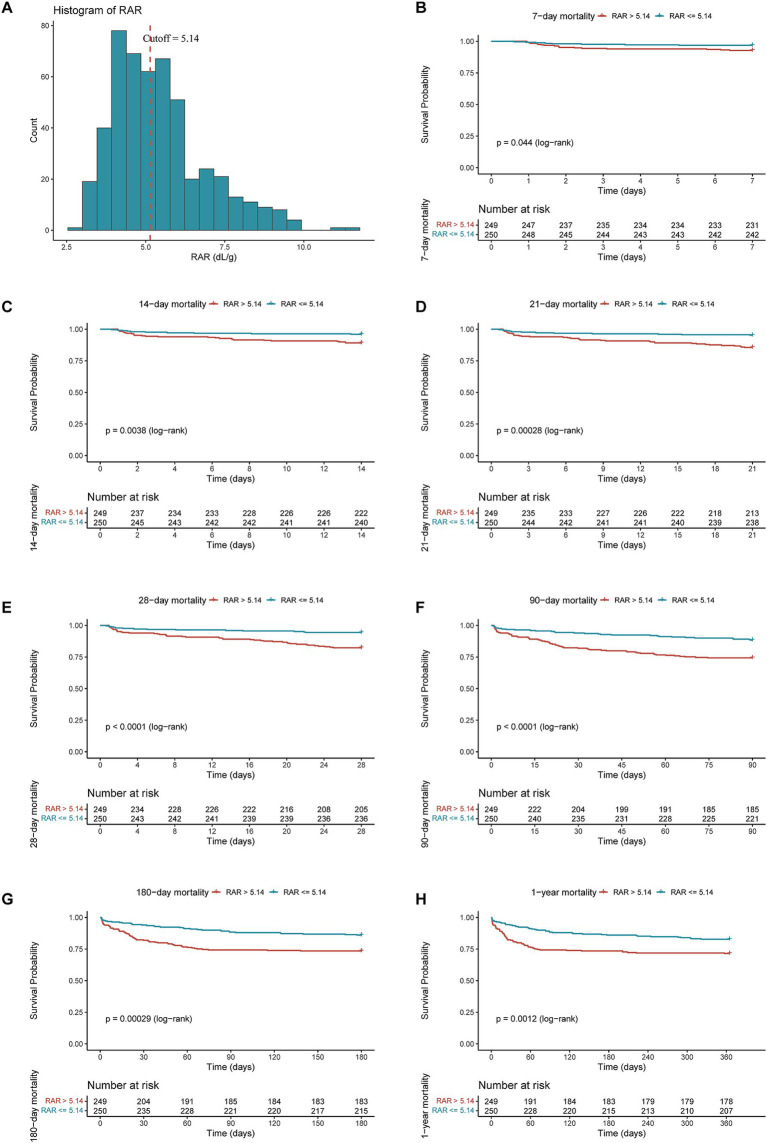
The cutoff point and distribution of RAR **(A)** and Kaplan–Meier survive curves for all-cause mortality at 7-d **(B)**, 14-d **(C)**, 21-d **(D)**, 28-d **(E)**, 90-d **(F)**, 180-d **(G)** and 1-y **(H)** after admission.

As illustrated by the K-M survival curves (Figure 2), compared with low-RAR group (RAR < = 5.14), high-RAR group (RAR > 5.14) had higher all-cause mortality at the 7-d (log-rank test *p* = 0.044), 14-d (log-rank test *p* = 0.0038), 21-d (log-rank test *p* = 0.00028), 28-d (log-rank test *p* < 0.0001), 90-d (log-rank test *p* < 0.0001), 180-d (log-rank test *p* = 0.00029), 1-y (log-rank test *p* = 0.0012).

Subsequently, univariate and multivariate Cox regression model were performed to reveal the latent relationship between RAR value and all-cause mortality in ICU patients with AP (Table 2). As a continuous variable, higher RAR was positively correlated with an increased risk of mortality at different timepoints in the unadjusted model: 28-d (HR = 1.53, 95% CI: 1.33–1.77, *p* < 0.001), 90-d (HR = 1.46, 95% CI: 1.30–1.64, *p* < 0.001), 365-d (HR = 1.38, 95% CI: 1.24–1.54, *p* < 0.001). After adjusting for age, gender and race (Model 1), higher RAR also represented an increased risk of mortality at different timepoints: 28-d (HR = 1.53, 95% CI: 1.32–1.77, *p* < 0.001), 90-d (HR = 1.49, 95% CI: 1.32–1.68, *p* < 0.001), 365-d (HR = 1.41, 95% CI: 1.26–1.58, *p* < 0.001). After adjusting for age, gender, race and other potential confounders which had *p*-value less than 0.1 in the univariate Cox regression model (Model 2), higher RAR still had a significant relationship with the increased risk of mortality at various periods: 28-d (HR = 1.63, 95% CI: 1.37–1.95, *p* < 0.001), 90-d (HR = 1.39, 95% CI: 1.21–1.59, *p* < 0.001), 365-d (HR = 1.31, 95% CI: 1.15–1.49, *p* < 0.001). After being transformed into a binary variable according to the median value of RAR, the results obtained from the univariate and multivariate COX regression model were still robust. The details are presented in the Table 2.

**Table 2:** Univariate and multivariate Cox regression models of CAR with mortality in patients with AP.

Categories	Unadjusted			Model 1			Model 2		
RAR	HR (95%)	*p*-value	*P* for trend	HR (95%)	*p*-value	*P* for trend	HR (95%)	*p*-value	*P* for trend
28-day mortality									
Continuous Variable	1.53 (1.33, 1.77)	<0.001		1.53 (1.32, 1.77)	<0.001		1.63 (1.37, 1.95)	<0.001	
Binary variable									
RAR ≤ 5.14			<0.001			<0.001			<0.001
RAR > 5.14	3.34 (1.83, 6.09)	<0.001		3.11 (1.70, 5.69)	<0.001		3.95 (1.98, 7.88)	<0.001	
90-day mortality									
Continuous Variable	1.46 (1.30, 1.64)	<0.001		1.49 (1.32, 1.68)	<0.001		1.39 (1.21, 1.59)	<0.001	
Binary variable									
RAR ≤ 5.14			<0.001			<0.001			0.002
RAR > 5.14	2.44 (1.57, 3.79)	<0.001		2.35 (1.51, 3.65)	<0.001		2.15 (1.32, 3.50)	0.002	
365-day mortality									
Continuous Variable	1.38 (1.24, 1.54)	<0.001		1.41 (1.26, 1.58)	<0.001		1.31 (1.15, 1.49)	<0.001	
Binary variable									
RAR ≤ 5.14			0.001			0.002			0.013
RAR > 5.14	1.85 (1.27, 2.70)	0.001		1.80 (1.23, 2.63)	0.002		1.72 (1.12, 2.62)	0.013	

Model 1: adjusted for age, gender and race at the above time points. Model 2 for 28-d mortality: adjusted for age, gender, race, Plt, K+, SOFA score, WBC, BUN, Cr, BG, Na+, TB, AF, AKI, CRRT, HF, MV, RF, sepsis and vasopressin. Model 2 for 90-d mortality: adjusted for age, gender, race, K+, SOFA score, WBC, ALP, BUN, Cr, Na+, PT, betablockers, CRRT, HF, MV, RF, sepsis and vasopressin. Model 2 for 1-y mortality: adjusted for age, gender, race, Plt, K+, SOFA score, WBC, ALP, BUN, Cr, PT, AF, AKI, betablockers, CRRT, HF, hypertension, MV, RF, sepsis and vasopressin. Plt, platelet; SOFA, Sequential Organ Failure Assessment; WBC, white blood cell count; BUN, blood urea nitrogen; Cr, creatinine; BG, blood glucose; TB, total bilirubin; AF, atrial fibrillation; AKI, acute kidney injury; CRRT, continuous renal replacement treatment; HF, heart failure; MV, mechanical ventilation; RF, respiratory failure; ALP, alkaline phosphatase.

### 3.3 RCS regression analysis

**Figure 3:**
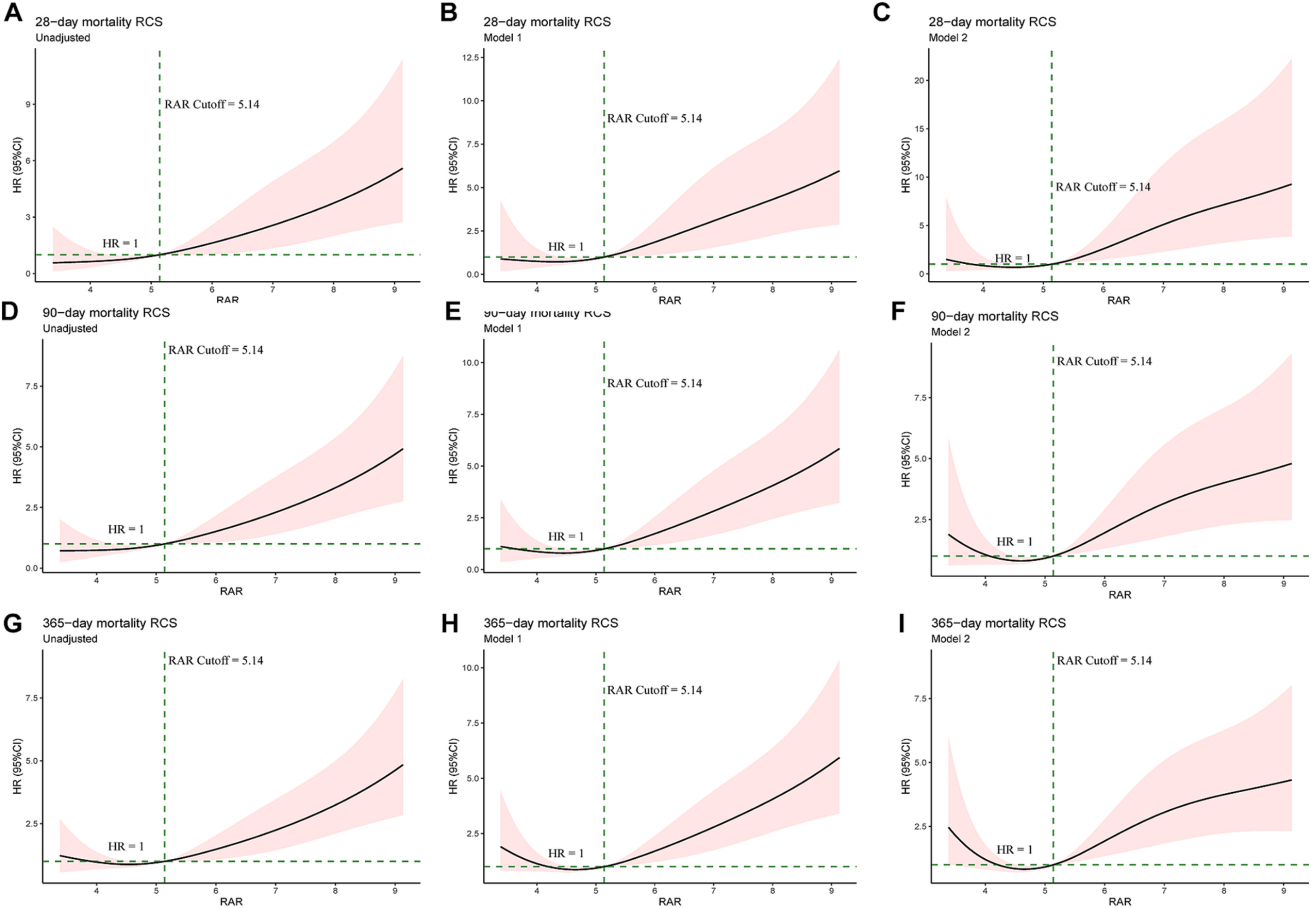
Univariate RCS regression showed the linear or non-linear association between the RAR and 28-d **(A–C)**, 90-d **(D–F)** and 1-y mortality **(G–I)**. Unadjusted RCS curves at the above time points (A, D and G). Model 1: adjusted for age, gender and race at the above time points **(B,E,H)**. Model 2 for 28-d mortality **(C)**: adjusted for age, gender, race, Plt, K^+^, SOFA score, WBC, BUN, Cr, BG, Na^+^, TB, AF, AKI, CRRT, HF, MV, RF, sepsis and vasopressin. Model 2 for 90-d mortality **(F)**: adjusted for age, gender, race, K^+^, SOFA score, WBC, ALP, BUN, Cr, Na^+^, PT, betablockers, CRRT, HF, MV, RF, sepsis and vasopressin. Model 2 for 1-y mortality **(I)**: adjusted for age, gender, race, Plt, K^+^, SOFA score, WBC, ALP, BUN, Cr, PT, AF, AKI, betablockers, CRRT, HF, hypertension, MV, RF, sepsis and vasopressin. Plt, platelet; SOFA, Sequential Organ Failure Assessment; WBC, white blood cell count; BUN, blood urea nitrogen; Cr, creatinine; BG, blood glucose; TB, total bilirubin; AF, atrial fibrillation; AKI, acute kidney injury; CRRT, continuous renal replacement treatment; HF, heart failure; MV, mechanical ventilation; RF, respiratory failure; ALP, alkaline phosphatase.

Exploratory RCS regression analysis were conducted to further investigate the potential non-linear relationship between RAR and adverse clinical outcomes. In the unadjusted RCS regression model, no significant linear relationship was observed between RAR and all-cause mortality at 28-d (*P* for non-linearity = 0.876), 90-d (*P* for non-linearity = 0.804) and 1-y (*P* for non-linearity = 0.141). After adjusting for age, gender and race (Model 1), no significant linear relationship was observed between RAR and mortality at 28-d (*P* for non-linearity = 0.575) and 90-d (*P* for non-linearity = 0.344) while there was a significant linear relationship between RAR and mortality at 1-y (*P* for non-linearity = 0.015). Based on model 1, after adjusting for other potential confounders (Model 2), which were variables with *p* value less than 0.1 in univariate Cox analysis, no significant linear relationship was observed between RAR and mortality at 28-d (*P* for non-linearity = 0.126) and 90-d (*P* for non-linearity = 0.064) while there was a significant linear relationship between RAR and mortality at 1-y (*P* for non-linearity = 0.007). Interestingly, in all the RCS models mentioned above, RAR became a risk factor (HR > 1) for all-cause mortality at 28-d, 90-d and 1-y when RAR exceeded 5.14. The details can be seen in Figure 3 and Supplementary Table 1.

### 3.4 Subgroup analysis

**Figure 4:**
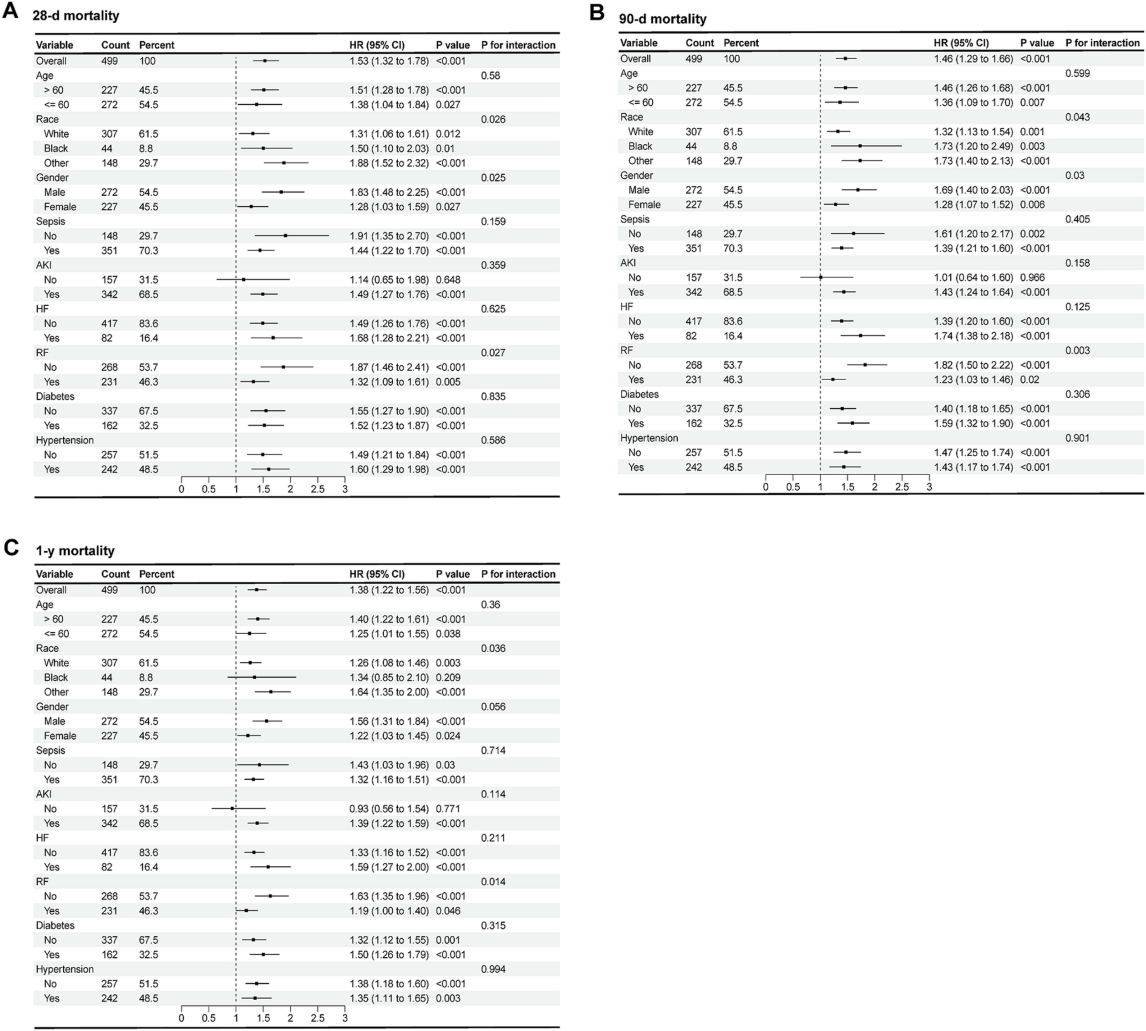
Forest plots of subgroup analysis of the relationship between all-cause mortality and RAR in patients with AP at 28-d **(A)**, 90-d **(B)**, 1-y **(C)** after admission.

As shown in Figure 4, the forest plots illustrated the presence of relationship between RAR and 28-d, 90-d and 1-y all-cause mortality in different groups of patients with AP. Stratified by age, gender, race, sepsis, AKI, heart failure (HF), RF, diabetes and hypertension, no significant interaction was observed between RAR and most subgroups (*P* for interaction >0.05), except for gender (*P* for interaction = 0.025), race (*P* for interaction = 0.026) and RF (*P* for interaction = 0.027). Compared with female patients with AP, male patients with elevated RAR had higher all-cause mortality at 28-d, 90-d and 1-y. Compared with white people, black people and patients from other races with increased RAR had higher death risk at 28-d, 90-d and 1-y. Furthermore, compared to the patients with RF, those without RF seemed to have higher all-cause mortality rate at 28-d, 90-d due to the incremental RAR value. The detailed results are presented in the Figure 4.

### 3.5 ROC analysis for all-cause mortality

**Figure 5:**
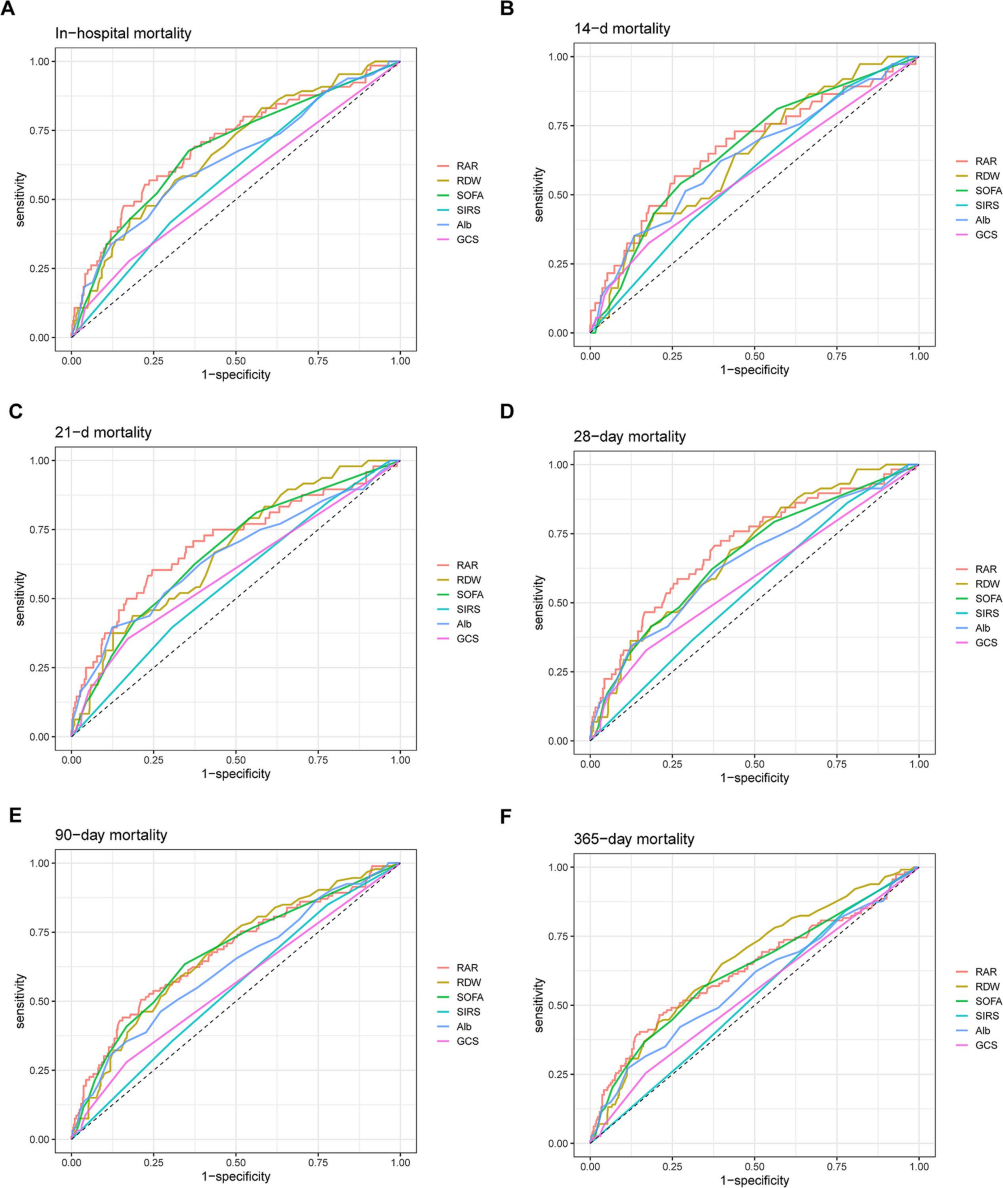
ROC curves for predicting in-hospital mortality **(A)** and all-cause mortality in patients with AP at 14-d **(B)**, 21-d **(C)**, 28-d **(D)**, 90-d **(E)** and 1-y **(F)** after admission.

**Figure 6:**
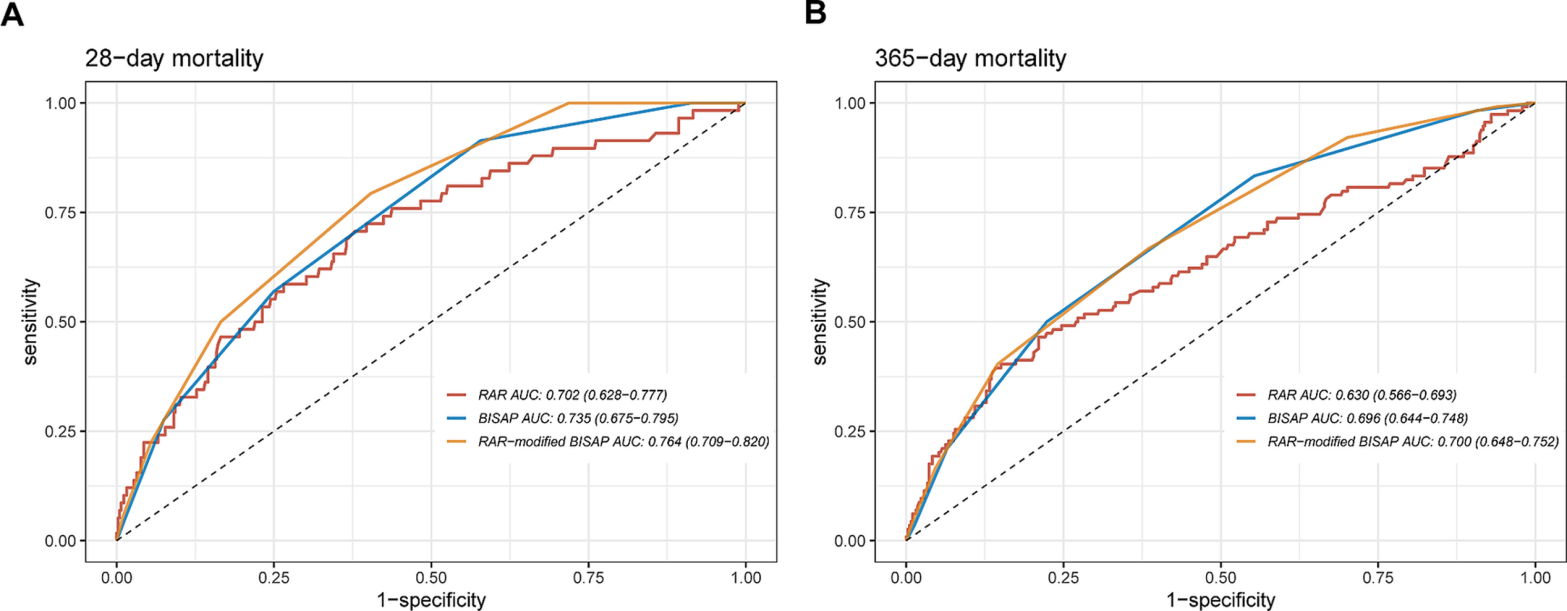
ROC curves for predicting all-cause mortality at 28-d **(A)** and 365-d **(B)** after combining RAR with BISAP.

ROC analysis was subsequently conducted to evaluate the predictive power of RAR for all-cause mortality of patients with AP. As illustrated by Figure 5 and Supplementary Table 2, in terms of AUC values for predicting all-cause mortality, RAR was superior to RDW, Alb, SOFA score, SIRS score and GCS score at 14-d, 21-d, 28-d, 90-d and in-hospital death, but not at 1-y. For instance, RAR (AUC: 0.701, 95%CI: 0.628–0.773) had greater predictive value for predicting in-hospital all-cause mortality than SOFA score (AUC: 0.682, 95%CI: 0.611–0.753), SIRS score (AUC: 0.585, 95%CI: 0.520–0.651), GCS score (AUC: 0.554, 95%CI: 0.495–0.613), RDW (AUC: 0.675, 95%CI: 0.607–0.774) and Alb (AUC: 0.644, 95%CI: 0.568–0.721). In terms of all-cause mortality at 28-d, compared with SOFA score (AUC: 0.666, 95%CI: 0.592–0.740), SIRS score (AUC: 0.551, 95%CI: 0.482–0.620), GCS score (AUC: 0.583, 95%CI: 0.518–0.647), RDW (AUC: 0.682, 95%CI: 0.613–0.751) and Alb (AUC: 0.647, 95%CI: 0.567–0.727), RAR had a higher diagnostic efficacy (AUC: 0.703, 95%CI: 0.628–0.777). Also, RAR had a slightly higher value (AUC: 0.674, 95%CI: 0.609–0.738) in predicting mortality at 90-d than SOFA score (AUC: 0.669, 95%CI: 0.608–0.731), SIRS score (AUC: 0.544, 95%CI: 0.485–0.602), GCS score (AUC: 0.557, 95%CI: 0.507–0.607), RDW (AUC: 0.671, 95%CI: 0.611–0.731) and Alb (AUC: 0.624, 95%CI: 0.559–0.689). Nevertheless, when predicting 1-y mortality, RAR (AUC: 0.630, 95%CI: 0.566–0.693) did not show any superiority to the above parameters. Subsequently, we added a RAR score into traditional BISAP scoring system. If RAR value exceed 5.14 (the median value), RAR score would be assigned as 1 otherwise it would be assigned as 0. As shown in Figure 6, compared with BISAP, RAR alone did not show any superiority in predicting short-term or long-term mortality. Interestingly, compared with BISAP (AUC: 0.735, 95%CI: 0.675–0.795) the RAR-modified BISAP exhibited an obvious improvement in forecasting 28-d mortality (AUC: 0.764, 95%CI: 0.709–0.820).

## 4 Discussion

Recently, a series of studies have investigated serum biomarkers such as the neutrophil/lymphocyte ratio (19), CRP/Alb ratio (11), lactate/Alb ratio (20) and creatinine/Alb ratio (21), to evaluate the prognosis of AP. RAR, as a novel biomarker derived from RDW and Alb, has been found to be associated with the severity of AP and participate in a nomogram which can predict the prognosis of AP (4, 17, 22). However, until now no research has been published on the value of RAR, as an independent factor, to predict the prognosis of AP. This study, based on our knowledge of the published literature, is the largest study examining the association of RDW with mortality in patients with AP.

Calculated on the basis of the mean red blood cell volume and its standard deviation, RDW is a common erythrocytic parameter which can be easily and quickly obtained from routine blood test (13). Its inexpensiveness and relatively low technical requirement mean that it is easily accessible in most medical institutions. RDW has long been regarded as an indicator to distinguish between thalassemia and iron deficiency anemia (13). In recent years, some studies have shown that RDW also has important predictive value in some non-hematological diseases, such as infectious diseases (23), autoimmune diseases (24) and cardiovascular diseases (25). For instance, based on a cohort consisting of 16,423 septic patients from multicenter eICU Collaborative Research Database, Dankl et al. (23) revealed that there was an association between mortality in sepsis and RDW, which showed a comparable diagnostic performance to SOFA score. Also, elevated RDW was found to be associated with severity and prognosis of AP. He et al. (15) analyzed 897 patients with AP from the MIMIC database and suggested that RDW was an independent risk factor affecting in-hospital mortality. Another retrospective study involving 42 patients with SAP revealed that RDW also has important predictive value for all-cause mortality in SAP, with an AUC greater than SOFA score (4). In this study, the AUC of RDW for predicting all-cause mortality in acute pancreatitis was similar to or slightly higher than the SOFA score, which is consistent with previous research reports. Possible mechanisms include inflammation of the pancreas, where a large amount of reactive oxygen species (ROS) produced by inflammatory cells, including superoxide anions, hydrogen peroxide, and hydroxyl radicals, directly damage the membrane of red blood cell, leading to the death of a large number of mature red blood cells and indirectly causing an increase in RDW. Various inflammatory factors produced by inflammatory cells, such as tumor necrosis factor alpha (TNFα) and interleukin 1 beta (IL-1β), can also activate related pathways leading to the apoptosis of erythrocyte (26). In addition, some researchers propose that inflammatory activity can inhibit iron metabolism and the production of erythropoietin (27). The decrease of erythropoietin hinders the maturation of red blood cells and promotes the release of immature cells into blood, ultimately leading to an increase in RDW levels.

As mentioned above, the pathogenesis of AP is closely related to oxidative stress and the activation of inflammatory response, which can lead to tissue damage. On the contrary, serum albumin promotes the production of several anti-inflammatory substances, including lipoproteins and lysins, which can help in wound healing and inflammation resolution (28). Consequently, a large amount of Alb is consumed during this process, which can partly explain the poor prognosis caused by the low Alb level in AP. However, serum albumin level is dynamically affected by multiple physiological and pathological factors such as chronic diseases, nutritional status and systemic inflammation, which may have limited predictive value in a single measurement (20, 21). In this study, Alb alone showed poor AUCs of predicting all-cause mortality in AP. Therefore, we analyzed the ratio of RDW to Alb which could reduce the influence of a single factor on the regulatory mechanism via the inverse changes caused by the two different factors, thereby more accurately predicting the prognosis of AP patients.

Previously, including 212 patients with mild acute pancreatitis (MAP) and 89 patients with SAP, a retrospective study suggested that in terms of predicting severity of AP, RAR (AUC: 0.884) had a comparable effectiveness with those of BISAP score (AUC: 0.922), Ranson score (AUC: 0.902) and MCTSI score (AUC: 0.928) (16). Moreover, Pan et al. (17) developed a nomogram based on RAR, age, heart rate, body temperature, AST/ALT, BUN, hemoglobin, potassium, and bilirubin to predict short-term all-cause mortality (30-d) in patients with acute pancreatitis. With RAR as the most significant laboratory indicator affecting prognosis, this novel scoring system was superior to other common scoring systems such as SOFA. However, there is currently no research that elucidates the predictive value of RAR for both short-term and long-term prognosis of pancreatitis, as well as how the associated risks vary when RAR values change. In this study, we firstly indicated that RAR was an independent risk factor affecting both short-term and long-term all-cause mortality in patients with AP. According to the survival curves, the short-term and long-term all-cause mortality rates of AP patients with RAR > 5.14 were significantly higher than those with RAR ≤ 5.14. Cox regression models and RCS regression analysis also suggested that RAR > 5.14 was an independent risk factor regardless of whether the potential confounders are adjusted or not. As mentioned before, RAR, as a composite indicator of RDW and Alb, can reflect the extent of local pancreatic necrosis and the overall nutritional status, which are related to some complications of pancreatitis such as sepsis, pancreatic fistula, pancreatic pseudocyst and abdominal compartment syndrome. This can partially explain the impact of RAR on short-term and long-term prognosis.

Subgroup analysis subsequently reveals that compared to other patients, elevated RAR is a more dangerous signal for male, non-white patients. We speculate that this is due to physiological differences across genders and genetic backgrounds across different races ethnic groups. Also, for those patients without respiratory failure, increased RAR levels bring higher risk of mortality. According to our speculation, this is because when patients with pancreatitis are accompanied by RF, the hypoxic state is exacerbated and the body generates more immature red blood cells and release them into the bloodstream compensatively, thereby RDW and RAR values rise. Under this circumstance, the explanatory power of RAR value for predicting all-cause mortality is weakened. This can be proven indirectly by some studies which suggest that elevated RDW and RAR are both independent risk factors affecting all-cause mortality in patients with RF (29, 30). Furthermore, the results of our study suggested that in terms of AUC values, RAR outperformed other control indicators in the in-hospital mortality and the short-term all-cause mortality (14-d, 28-d and 90-d). SIRS and GCS are classic scoring systems for evaluating the degree of systemic inflammatory response and the consciousness status of critically ill patients. However, in AP, they may not be reliable indicators due to the complex pathophysiological characteristics. For other scoring systems like SOFA, they consist of multiple items and require continuous or repeated observations, which means that medical staffs cannot accurately assess the patients’ condition at admission. Due to the easy availability and low cost of RAR, it may be more suitable for primary healthcare institutions to quickly assess the prognosis and severity of pancreatitis, and to promptly transfer patients for further treatment. Notably, according to our results, RAR was not superior to RDW alone in predicting 1-y mortality although its performance was greater than that of first SOFA score in ICU. The reason might be that when predicting long-term prognosis of patients with AP, Alb is more affected by other factors such as patients’ nutritional status in recovery period, which increases the instability of the predictive power of RAR overall. We also tried to use RAR to modified the traditional BISAP scoring system. After being added a RAR score, the modified BISAP score exhibited improvement in predicting short-term mortality but did not show obvious advantage in long-term mortality prediction. This finding emphasized the value of RAR in predicting short-term mortality. Unfortunately, due to the complexity of Ranson score, we did not obtain complete data to calculate Ranson score and compared RAR with it.

Certainly, our results still have some limitations. Firstly, we conducted our study on the relationship between the first RAR and prognosis after ICU admission, which made it impossible for us to evaluate the impact of dynamic RAR on prognosis. Secondly, in order to obtain a greater sample size and get a conclusion generalized to more patients in ICU, we did not strictly limit the inclusion criteria for AP to the first diagnosis, which may cause some biases. What is more, our study is a single center retrospective cohort study and cannot fully elucidate the relationship between RAR and AP like prospective studies, leading to the lack of enough persuasiveness. Finally, the population data we studied comes from MIMIC-IV (v3.0), which covers hospitalized patients from 2008 to 2022. With the development of medical treatment and optimization of treatment plans, such a long period of time cannot guarantee the consistency of treatment plans for hospitalized patients.

## 5 Conclusion

In our study, RAR > 5.14 is an independent risk factor for both short-term and long-term all-cause mortality. RAR could serve as a prognostic predictor of short-term all-cause mortality and in-hospital mortality, which performs better than RDW and Alb alone and is not inferior to SOFA score. For patients with AP, once RAR increases, male, non-white and patients without AP have a greater risk of death.

## Data Availability

Publicly available datasets were analyzed in this study. This data can be found at: https://mimic.mit.edu/.

## Footnotes

^1^ https://mimic.mit.edu/
