# Supplementary material for: Association between red cell distribution width to albumin ratio and all-cause mortality in patients with acute pancreatitis admitted to the intensive care unit: a retrospective study based on the MIMIC-IV database
Source: Front Med (Lausanne). 2025 Feb 12;12:1503378. doi: 10.3389/fmed.2025.1503378 (PMC11863955; doi:10.3389/fmed.2025.1503378)
Supplement: Supplementary file 1 [file Table_1.docx]

Supplementary Material

# Supplementary tables

## Supplementary table 1 The *P*-values of linearity test in RCS

| Category | Unadjusted | Model 1 | Model 2 |
| --- | --- | --- | --- |
| 28-day mortality | | | |
| P for non-linearity | 0.876 | 0.575 | 0.126 |
| 90-day mortality | | | |
| P for non-linearity | 0.804 | 0.344 | 0.064 |
| 1-y mortality | | | |
| P for non-linearity | 0.141 | 0.0146 | 0.007 |

## Supplementary table 1 The results of ROC analysis

| Variables | AUC | 95%CI |
| --- | --- | --- |
| In-hospital mortality | | |
| RAR | 0.701 | 0.628-0.773 |
| RDW | 0.675 | 0.607-0.744 |
| Alb | 0.644 | 0.568-0.721 |
| SOFA | 0.682 | 0.611-0.753 |
| SIRS | 0.585 | 0.520-0.651 |
| GCS | 0.554 | 0.495-0.613 |
| 14-d mortality | | |
| RAR | 0.671 | 0.572-0.770 |
| RDW | 0.645 | 0.556-0.733 |
| Alb | 0.637 | 0.538-0.736 |
| SOFA | 0.664 | 0.576-0.752 |
| SIRS | 0.578 | 0.494-0.661 |
| GCS | 0.579 | 0.497-0.660 |
| 21-d mortality | | |
| RAR | 0.702 | 0.615-0.789 |
| RDW | 0.673 | 0.596-0.749 |
| Alb | 0.653 | 0.562-0.743 |
| SOFA | 0.669 | 0.590-0.747 |
| SIRS | 0.562 | 0.484-0.639 |
| GCS | 0.596 | 0.523-0.668 |
| 28-d mortality | | |
| RAR | 0.703 | 0.628-0.777 |
| RDW | 0.682 | 0.613-0.751 |
| Alb | 0.647 | 0.567-0.727 |
| SOFA | 0.666 | 0.592-0.740 |
| SIRS | 0.551 | 0.482-0.620 |
| GCS | 0.583 | 0.518-0.647 |
| 90-d mortality | | |
| RAR | 0.674 | 0.609-0.738 |
| RDW | 0.671 | 0.611-0.731 |
| Alb | 0.624 | 0.559-0.689 |
| SOFA | 0.669 | 0.608-0.731 |
| SIRS | 0.544 | 0.485-0.602 |
| GCS | 0.557 | 0.507-0.607 |
| 1-y mortality | | |
| RAR | 0.630 | 0.566-0.693 |
| RDW | 0.657 | 0.600-0.714 |
| Alb | 0.583 | 0.520-0.646 |
| SOFA | 0.626 | 0.566-0.686 |
| SIRS | 0.528 | 0.474-0.582 |
| GCS | 0.543 | 0.498-0.587 |
